# Progression of Liver Diseases

**DOI:** 10.5812/hepatmon.6626

**Published:** 2012-06-30

**Authors:** Miriam Liliana Cuarterolo

**Affiliations:** 1 Hepatology Unit, Hepatology Hospital de Pediatría Juan P. Garrahan, Buenos Aires, Argentina

**Keywords:** Liver, Disease, Hepatitis C

Dear Editor,

To date, the knowledge on the natural history of HCV infection and the risk factors associated with progression of liver disease in children is limited. Progressive liver disease, including hepatocellular carcinoma, is rare but has been reported [1]. Early effective treatment can prevent potential hepatic complications in later life. The association of Peg-IFN plus ribavirin is the option of choice for the treatment of HCV infection in children [2]. Patients treated with this therapeutic scheme showed a sustained virological response (SVR) rate of 65 %, being genotype the main predictor of treatment outcome (53 %, 93 %, 93 %, and 80 % with genotype 1, 2, 3, and 4, respectively [3]. Several important factors, such as genotype except genotype 1 and low pretreatment serum HCV RNA levels, are associated with favorable therapeutic response. Rapid virological response and early virological response are strong predictors of treatment outcome in genotype 1 patients [3]. Mild fibrosis, present in the biopsy of the majority of children with this infection, may also contribute to higher rates of SVR. Adverse effects, particularly those related to mild leukopenia, neutropenia, and thrombocytopenia, are frequently observed during treatment [2]. Low hemoglobin levels may be resulted from ribavirin-induced hemolysis or from IFN-induced bone marrow suppression. Sulkowski et al. [4] have recently showed in a large retrospective study, that in treatment of-naïve HCV genotype 1 adult patients treated with Peg-IFN plus ribavirin, patients with early-onset anemia (≤ eight weeks of treatment) had higher SVR rates with erythropoiesis-stimulating agents, whereas no effect was observed in whom with late-onset anemia.

Similar results were obtained by Sievert et al. [5]. They found that in patients with HCV genotype 1 infection, the odds for achieving SVR for those whom lowest hemoglobin was < 100 g/l or whom maximum hemoglobin decline was 30 g/L, were about twice the odds of those whom maximum hemoglobin decline was ≤ 30 g/L or those whom lowest hemoglobin during treatment was ≥ 100 g/L. The mechanisms underlying the higher SVR rates in patients with a decline in hemoglobin remain unclear. The article published by Malgorzata et al. is of great interest because of its focus on the relationship between the hematological adverse effects and the response to therapy in children with chronic hepatitis C. 170 patients were included in the study and divided into two groups according to the treatment scheme. Group I (n: 119) received recombinant IFN-α-2b plus RBV and group II (n: 51) was administered peg- IFN-α-2b plus ribavirin. Both groups were treated with a 48-week course. Hematological growth factors and erythropoietin were not used. The authors showed a better response to treatment in terms of obtaining SVR in those patients who presented more hematological adverse effects. With ongoing research, such as this study by Malgorzata et al., investigating markers of response to treatment, great advances can be made for children who will be infected with HCV over the next several years.
